# The Influence of Supercritical Carbon Dioxide on Graham Flour Enzyme Polyphenol Oxidase Activity

**DOI:** 10.3390/molecules25245981

**Published:** 2020-12-17

**Authors:** Gordana Hojnik Podrepšek, Željko Knez, Maja Leitgeb

**Affiliations:** 1 Laboratory for Separation Processes and Product Design, Faculty of Chemistry and Chemical Engineering, University of Maribor, Smetanova ulica 17, 2000 Maribor, Slovenia; gordana.hojnik@um.si (G.H.P.); zeljko.knez@um.si (Ž.K.); 2 Faculty of Medicine, University of Maribor, Taborska ulica 8, 2000 Maribor, Slovenia

**Keywords:** graham flour, supercritical CO_2_, polyphenol oxidase, enzyme inactivation

## Abstract

Graham flour is a form of whole wheat flour made by grinding the endosperm and is thus also the most nutritious. Generally, the enzyme polyphenol oxidase (PPO) catalyzes two different reactions in the presence of molecular oxygen: the hydroxylation of monophenols to ortho-diphenol and the oxidation of *o*-diphenol to *o*-quinone. The purpose of the work was to inactivate PPO activity to extend the shelf life of graham flour and at the same time preserve all the of its high-quality properties. The influence of supercritical CO_2_ (scCO_2_) treatment on PPO activity in graham flour was investigated. First, graham flour was exposed to scCO_2_ conditions, then the proteins were extracted, and in the last step the concentration of total proteins and the specific activity of the PPO enzyme were determined by spectrophotometric assay. PPO activity decreased with an increase in treatment pressure. Furthermore, the flour quality characteristics that meet all needs for wheat end-use products after scCO_2_ treatment have been preserved. No major changes in the structure of the granulate or shape of the flour particles were observed. A slightly reduced value of the moisture content in scCO_2_-treated graham flour also implies an extension of the shelf life.

## 1. Introduction

Graham flour is similar to whole wheat flour, as it contains the germ, endosperm, and bran of the wheat berry. Products vary in granulation and percentage content of the whole kernel, and the size of the bran particles affects the baking quality of the mix [1]. Moreover, the quality of flour is also affected by enzymes that are naturally present in flour. One of these is enzyme polyphenol oxidase (PPO), which catalyzes the oxidation of *o*-diphenols to produce *o*-quinones and is involved in food browning. This browning causes the deterioration of food, causing large economic losses. Moreover, a recent study from Zhai et al. also discovers the importance of PPO enzyme in flour onto time-dependent discoloration and darkening of wheat (*Triticum aestivum* L.) products. Based on the results obtained, it was established that PPO activity in flour is the main cause of undesirable darkening of wheat end-use products [2]. Enzymatic browning has been studied extensively in fruits and vegetables [3,4], and has also been implicated in the discoloration, called melanosis or blackspot, in shrimp [5].

**Figure 1:**
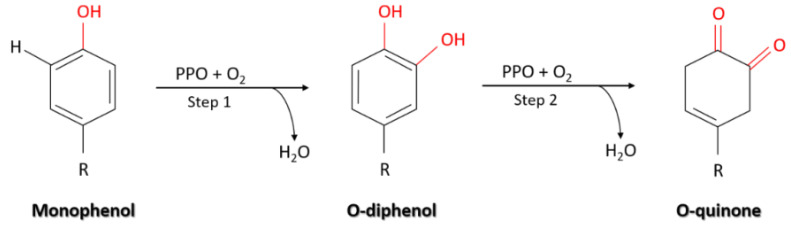
Schematic illustration of the reaction mechanism catalyzed by polyphenol oxidase (PPO).

PPO is a dicopper-containing enzyme involved in oxidation of the polyphenols [6]. Generally, PPO catalyzes two different reactions in the presence of molecular oxygen: the hydroxylation of monophenols to ortho-diphenols and further oxidation of *o*-diphenol to *o*-quinone [6,7,8]. The schematic reaction catalyzed by PPO is presented in Figure 1.

Enzymatic browning has also been implicated in the discoloration of flour, an issue that has already attracted the attention of some researchers. A heat treatment procedure is often applied for enzyme inactivation with the use of a microwave oven operated at 900 W for 80 s, as the influence of high temperatures above 50 °C could cause several biological, chemical, and physical modifications leading to sensory, nutritional, and textural changes, affecting both the quality and biocomponents of the flour [9]. Based on the fact that it is difficult to inactivate PPO by heat treatment without destroying gluten [10], where details are also described in the study of Zhao et al. [11], the current study is important because the technology with scCO_2_ used for inactivation of PPO activity in graham flour is suitable as it does not adversely affect the quality of the flour. Furthermore, the latest study from Guo et al. presented the achievement of high inactivation of the PPO enzyme using the superheated steam treatment procedure in which a very high temperature of up to 190 °C was used [12]. If we compare scCO_2_ technology, it is much more suitable as throughout the whole procedure. The temperature was only 35 °C, which is an additional parameter, which proves that with the scCO_2_ technology the properties of flour remain the same as those of untreated flour.

The application of scCO_2_ has already been shown to be suitable for enzyme inactivation in our previous research, due to its specific characteristics. Dense carbon dioxide in the supercritical state is nontoxic and non-flammable [13], and thus suitable for treatment of food. In this article, scCO_2_ was used for inactivation of PPO in graham flour in order to prolong the shelf life of the flour and prevent the browning process, which occurs in the presence of the enzyme PPO. For this reason, a specific non-thermal technology with scCO_2_ was used for the inactivation of enzyme PPO in graham flour. High-pressure carbon dioxide (HPCO_2_) has previously been reported as a promising non-thermal technology for the stabilization of fresh products. During such treatment, food is in contact with pressurized CO_2_ [14]. Moreover, the conditions when using scCO_2_ are above the critical temperature and critical pressure (31.1 °C, 73.8 bar) [15,16]. Further, CO_2_ is an environmentally friendly solvent that is ideal for food processing [17,18]. Typical CO_2_ pressure is generally within 40 and 300 bar, rarely exceeding 500 bar. The temperature is generally much lower than that seen with conventional treatments, between 20 °C and 50 °C, low enough to represent a non-thermal treatment [19]. It has been demonstrated that the activity of enzyme PPO in various foods (red beet, apple, potatoes, mushrooms, grape juice, radish, red raspberry, strawberry, and pineapple) decreased after thermal treatment [20], high hydrostatic pressure [21], HP CO_2_ treatment [19,22,23,24,25], ohmic heating [26], ultrasound treatments [27], and treatment with subcritical/scCO_2_ [28]. Yadav et al. presented a study in which PPO activity in wheat dough decreased significantly after microwave heating, where a 93.39% reduction in PPO activity was achieved in the whole wheat flour by microwave heating of wheat grains for 80 s at 18% moisture content [29]. Nonetheless, in order to maintain the quality of the flour, it is preferable to use a non-thermal treatment with a lower temperature to achieve inactivation of PPO.

For the first time, the detailed influence of a sustainable process using scCO_2_ on graham flour is presented in the current study. The article describes inactivation of the enzyme PPO in graham flour, where the activity of PPO was affected by scCO_2_ treatment under different conditions. Moreover, the structural properties of untreated and scCO_2_-treated graham flour were characterized using scanning electron microscopy (SEM), a particle size analyzer and moisture content analyzer.

## 2. Results and Discussion

### 2.1. Determination of Protein Concentration in Graham Flour

**Figure 2:**
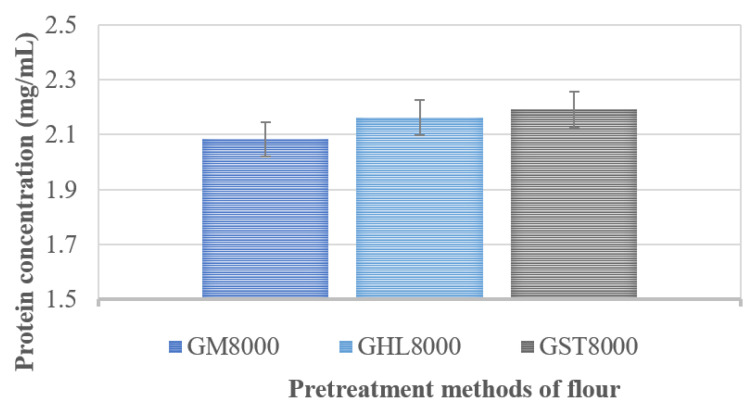
Pretreatment methods of flour influence on protein concentration; GM8000—graham flour prestored at room temperature, GHL8000—graham flour prestored in refrigerator at 4 °C, and GST8000—graham flour prestored at room temperature with glass beads.

The effects of flour pre-storage on the protein concentration of the flour were determined by different methods of pre-preparation. More specifically, glass beads were added to the graham flour before the extraction process, where a slightly better extraction was expected since glass beads can provide efficient disruption, homogenization, and high yields of extracted flour proteins [30]. Protein extraction was carried out, and differences in the concentrations of total proteins in the supernatants after extraction were examined. As a result, three different methods of flour treatment were used in the optimization of the extraction process: flour prestored at room temperature (GM8000), flour prestored at 4 °C (GHL8000), and flour prestored at room temperature with the addition of glass beads (GST8000) at a centrifugation speed of 8000 rpm. The centrifugation time was 5 min. It can be seen from Figure 2 that there is a slight difference in the protein concentration in the supernatants, as the protein concentration in these methods strongly depends on the extraction conditions.

Figure 2 also shows that the highest protein concentration of 2.2 mg/mL was determined in the supernatant after extraction of the proteins in flour with the addition of glass beads during the extraction process. Moreover, the concentration of extracted proteins was higher when the flour was prestored in a refrigerator at 4 °C, achieving 2.16 mg/mL, compared to that seen with flour prestored at room temperature, with a protein concentration of 2.08 mg/mL.

Pre-storing flour at 4 °C positively affected the thermal stability of the enzyme, resulting in a higher protein concentration after protein extraction from graham flour. The use of glass beads also enhanced the disruption of the protein matrix and facilitated better access to protein molecules [31], which was reflected in the highest concentration of proteins that were extracted from flour.

Based on these results, the optimal method with the highest protein concentration is a process where glass beads have been added to the flour, and therefore we decided to use this method for extraction of proteins from graham flour in further tests.

### 2.2. Protein Concentration after scCO_2_ Treatment

**Figure 3:**
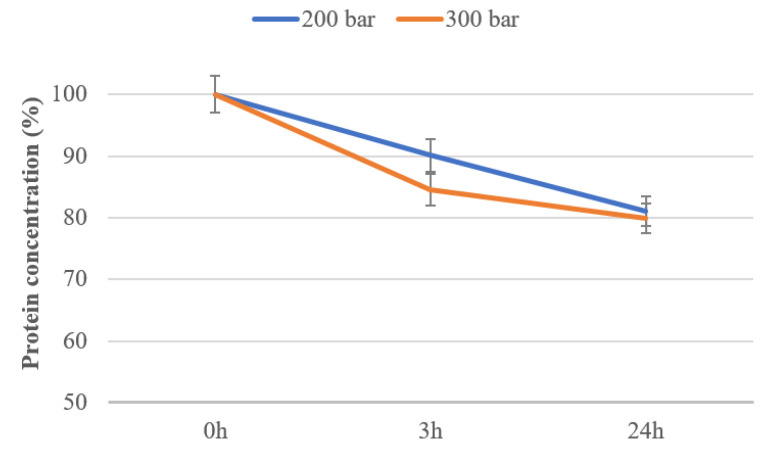
Comparison of the protein concentration of untreated (0 h) and scCO_2_-treated graham flour at 200 bar and 300 bar after treatment times of 3 and 24 h.

The protein concentration after protein extraction from graham flour was determined by the Bradford method. The effect of scCO_2_ on protein concentration was evaluated after graham flour exposure to scCO_2_ at different conditions, with the results presented in Figure 3. Furthermore, the protein concentration in untreated flour was taken for comparison as an initial value. Figure 3 presents the protein concentration in untreated and scCO_2_-treated graham flour. As can be seen from the results, the protein concentration in graham flour decreased after scCO_2_ exposure. Additionally, with increasing pressure it is observed that the protein concentration was reduced by a maximum of 20% after scCO_2_ treatment for 24 h and 300 bar, confirming that scCO_2_ induced protein loss in graham flour. The protein loss in scCO_2_-treated graham flour in this study is mainly ascribed to leakage of proteins into the media. A similar effect was also described in Liao et al. (2010) [32], where the protein content decreased after HPCO_2_ treatment of *E. coli* cells by extending the exposure time.

### 2.3. PPO Inactivation under scCO_2_ Treatment

**Figure 4:**
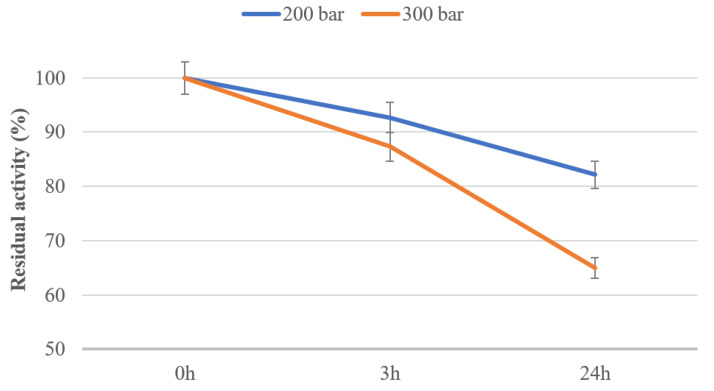
Residual activity of PPO in graham flour after exposure to scCO_2_ at 200 bar and 300 bar after treatment times of 3 and 24 h.

After determination of the protein concentration in supernatant, obtained by extraction after centrifugation, the PPO activity of untreated and scCO_2_-treated graham flour was determined, as described in Section 3.6. The influence of scCO_2_ on the residual activity of the PPO enzyme in graham flour at different pressures and with different treatment times at 35 °C is presented in Figure 4. The results show that PPO activity decreased with an increase of treatment pressure. A 13% decrease in PPO activity already occurred after 3 h at 300 bar, so the residual activity of PPO after 3 h of scCO_2_ treatment of graham flour at 300 bar was 87%. Moreover, after 24 h of treatment at 200 bar there was an 18% decrease in PPO activity, and after scCO_2_ treatment at 300 bar the residual activity of PPO decreased significantly to 65% of the initial value of the untreated flour. Similar to the results for protein concentration, at a higher pressure a greater inactivation of the PPO in graham flour was achieved. As the residual activity of PPO in graham flour was lower at higher pressure (300 bar), it is obvious that a pressure of 300 bar for scCO_2_ treatment is more suitable for the inactivation of PPO.

The effects of pressure in a supercritical fluid environment are complex and can be divided into three categories. First, the direct effects of pressure on the conformation of enzyme molecules (stability); second, the effects on intrinsic enzyme kinetics; and third, the effects on the physical properties of supercritical fluids, which can then indirectly modulate enzyme activity, specificity, and stability [33].

These observations coincide with the work of Marszałek et al. (2019), which explains the sensitivity of the PPO enzyme to scCO_2_ pressure and temperature. This earlier study describes inactivation of PPO from *Agaricus bisporus*, where the residual activity of PPO after scCO_2_ treatment at 300 bar and 45 °C was 60%. With prolongation of the time (30 min), higher pressure (650 bar), and higher temperature (50 °C), the lowest PPO residual activity of 12% was obtained [34]. However, it should be noted that the results are not equally comparable to ours, as in Marszałek et al. the microbial PPO enzyme was used, whereas our study describes the effects of scCO_2_ on the PPO enzyme in graham flour. Furthermore, in another study, Marszałek et al. (2017) described the inactivation of PPO in beetroot juice, which showed that the rate of decrease in PPO activity after scCO_2_ treatment was very low, only a few percent, although a high pressure of 600 bar and high temperature were used [35].

Our study was focused on lower pressure conditions compared to those in earlier works due to possible changes in flour quality [36] (in granulation, lipid content, flour moisture, etc.). Therefore, only pressure up to 300 bar was used for flour treatment, in order to preserve all the valuable characteristics of the flour, because scCO_2_, under these conditions had no deteriorating effects on its nutritional value [37], while at the same time seeking the greatest decrease in PPO activity possible, as this activity causes unwanted processes in the context of long-term flour storage.

### 2.4. The Effects of scCO_2_ on Total Phenolic Contents

The results of total phenolic content in untreated graham flour compared to scCO_2_-treated graham flour at 200 and 300 bar for 3 h revealed that the polyphenols content increased during scCO_2_ treatment. The polyphenols content expressed in mg GA/g flour was 0.036 for untreated graham flour, while that of graham flour treated with scCO_2_ at 200 bar was 0.037 and at 300 bar it was 0.038. In a study, Hironaka et al. (2006) found that the polyphenol content in potato correlated with PPO activity during storage [38]. Moreover, Esmaeili et al. (2017) showed an inverse correlation between phenolic content and PPO activity [39], which is consistent with the results of the current study.

### 2.5. The Effects of scCO_2_ on Graham Flour Properties

In order to determine the influence of scCO_2_ on graham flour quality, characterization of flour particles was performed by SEM and particle size analyses. In addition, the moisture content for both untreated and scCO_2_-treated graham flour was examined.

**Figure 5:**
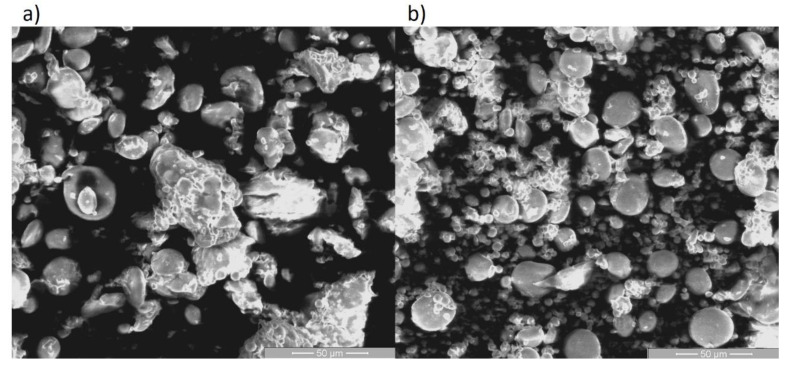
SEM micrographs of graham flour: (**a**) untreated and (**b**) scCO_2_-treated at 300 bar and 3 h at 1000× magnification.

The mean particle size and shape of the untreated and scCO_2_-treated graham flour were determined. SEM micrographs of the untreated and scCO_2_-treated graham flour at the same magnification level are shown in Figure 5. In general, no major changes in granulate structure and flour particle form were observed, and round starch granules with smooth surfaces were identified in both treated and untreated graham flour. According to the SEM analysis, the flour particles of untreated graham flour were distributed within the size range of 22 to 116 μm, while the flour particles of the scCO_2_-treated graham flour were within the size range 16 to 83 μm. Moreover, it can be observed that larger clusters of particles disintegrated after scCO_2_ treatment, which is consistent with the particle size measurement results, as presented in the Table 1. The results presented in Figure 5 clearly demonstrate that scCO_2_ does not adversely affect the quality of the flour, nor does it not significantly change the structure. This provides further support for the idea that scCO_2_ treatment is appropriate for preprocessing of graham flour, which could be further used in the bakery industry.

Moisture content affects the processibility, shelf life, usability, and quality of the final graham flour product. Moisture content determination therefore plays a key role in ensuring quality with regard to applications in the food industry. Consequently, an analysis of the moisture content in both untreated and scCO_2_-treated graham flour was conducted to determine if scCO_2_ affects flour quality. Table 1 indicates that there was no significant difference in the moisture content of the samples. The 0.5% decrease of the moisture content in scCO_2_ treated graham flour even indicates an extension of the shelf life of the flour. On the other hand, the moisture content was not so reduced to affect further flour processing in the manufacture of bakery products in terms of its mechanical, thermal, and rheological properties [40]. It is also considered that a moisture content over 14% attracts the growth of mold, bacteria, and various insects, which can damage the flour quality during storage. Lapčíková, et al. considered impact of the particle size of a variety of flours on the baking properties of dough. The water contents in the dough and bread made from variety of white and brown (containing bran particles) flours with different particle size distributions were analyzed. The findings showed that the quality of the resulting bread decreased due to the large particles in coarse flour, and that flour with fine particles exhibited a high ability to elongate and accumulate stress in the dough prepared from it, where the gluten proteins, starch and other substances were released to a greater extent from the flour particles into the dough [41].

The properties of scCO_2_-treated graham flour changed slightly, and it became lighter due to the reduced moisture content. As a result, certain aggregates disintegrated, which is reflected in the reduced value of the particle size distribution. In short, the scCO_2_ medium successfully inactivated the enzymes and at the same time did not weaken the key properties of the flour that are crucial in the production of bakery products.

## 3. Materials and Methods

### 3.1. Reagents

Graham flour was kindly donated by the bakery Hlebček d.o.o. (Pragersko, Slovenia). Analyses were conducted using carbon dioxide (99.5% purity) produced by Messer, Ruše. Ethanol, phosphoric acid, and Coomassie-Brilliant Blue G250 were supplied from Merck (Darmstadt, Germany), while bovine serum albumin (BSA), sodium acetate, potassium phosphate, l-3,4-dihydroxyphenylalanine, l-ascorbic acid, and ethylenediaminetetraacetic acid were supplied from Sigma (Steinheim, Germany). All other chemicals used in the laboratory were of analytical grade.

### 3.2. Overview of Experimental Procedure

**Figure 6:**
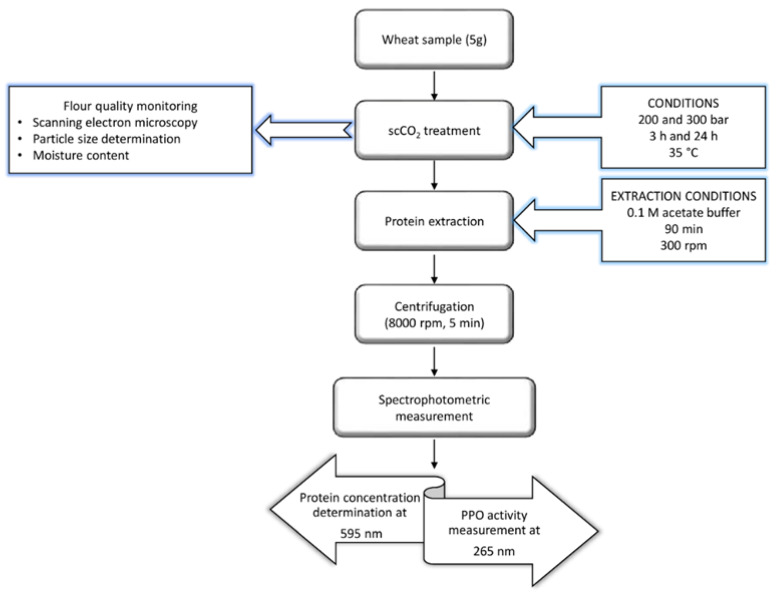
Flow diagram showing the procedure of graham flour analysis.

An overview of the experimental procedure in Figure 6 describes the experimental setup and treatment studies for graham flour analysis. The graham flour sample was first exposed to scCO_2_ under the described conditions. The process of exposure was followed by protein extraction, where the resulting clear supernatant was used for spectrophotometric measurements of PPO activity and protein determination in graham flour.

### 3.3. Supercritical CO_2_ Treatment

**Figure 7:**
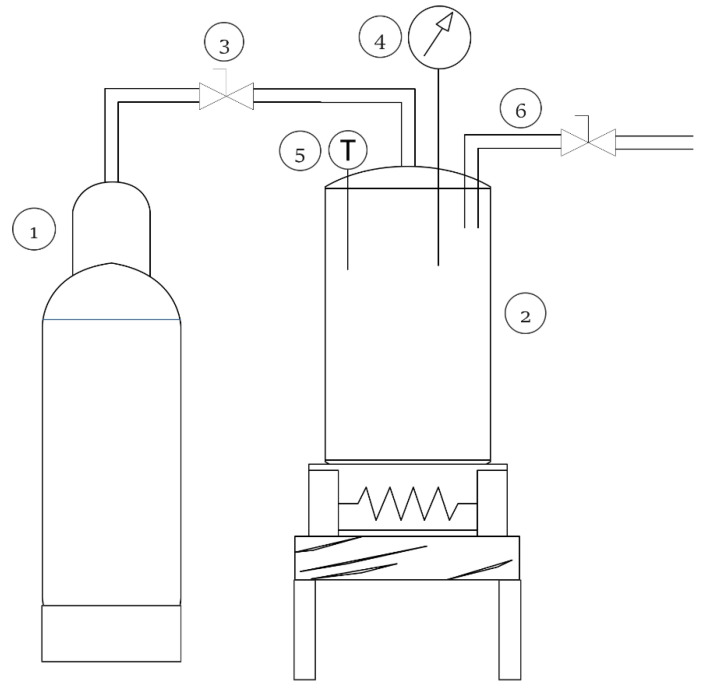
Supercritical carbon dioxide reactor scheme: 1: CO_2_ cylinder, 2: high pressure reactor, 3: valve, 4: pressure indicator, 5: temperature indicator, and 6: CO_2_ release.

Graham flour was exposed to scCO_2_ in a 60 mL high-pressure batch reactor at 35 °C to the desired pressure (200 bar and 300 bar) for a defined time, with the scCO_2_ system shown in Figure 7. When the temperature in the high-pressure batch reactor with graham flour reached 35 °C, cooled CO_2_ was added to the reactor until the desired pressure was obtained. Afterwards, the reactor was quickly depressurized (Δp/Δt = 0.37 ± 0.08 bar/min). Furthermore, a sample of flour was taken out of the reactor and left at room temperature for some time to ensure that CO_2_ was released out of the sample. The enzyme activity of the treated samples of graham flour was compared with that of the untreated graham flour.

It is known that exposure of enzymes to higher temperatures leads to their inactivation [42]. As the optimum temperature for the PPO enzyme activity is close to 30 °C, a temperature of 35 °C was chosen as the operating temperature for the experiments to avoid inactivation of the enzymes caused by the higher temperature and allow the CO_2_ to reach its supercritical state. All experiments were done in triplicate and the error bar represents the percentage error (±3%) in each set of readings.

### 3.4. Optimization of Protein Extraction from Graham Flour

Proteins were extracted from graham flour (5 g) with 0.1 M acetate buffer solution (pH 5.3) by shaking for 90 min at room temperature. To optimize the process of protein extraction from graham flour and obtain the best possible yield, various parameters, which are specifically listed in Table 2 below, were used. To ensure efficient disruption, homogenization, and higher concentration of protein from flour, glass beads were added to the mixture. The flour was also kept in a refrigerator to find out if a higher protein concentration could be achieved by extracting proteins from flour stored between 2 and 8 °C. The suspension was then centrifuged at a speed of 8000 rpm for 5 min, and the clear supernatant was collected. The concentration of proteins was determined in supernatant samples using the Bradford protein assay method [43].

### 3.5. Determination of Protein Concentration

Protein concentration was determined according to the Bradford method using BSA as the standard protein [43], where a standard curve of BSA was obtained, ranging from 0 to 1 mg/mL. The protein concentration was determined by direct measuring of the protein content in the remaining supernatant obtained by the protein extraction process.

The absorbance was measured in the supernatant after centrifugation using a colorimetric method and a UV-spectrophotometer with the wavelength set at 595 nm (Varian Cary Probe 50, Agilent Technologies, Santa Clara, CA, USA). Each sample was assayed twice and three individual absorbance measurements per extracted sample were recorded. Moreover, the protein concentration *c* (mg/mL) in the samples was calculated using the standard curve of BSA from Equation (1),

(1) $$c=\frac{A_{595\;\mathrm{nm}}}{k}\;\left(\frac{mg}{mL}\right)$$

where *A*_595 nm_ is the sample absorbance measured at 595 nm, and *k* is the slope of the calibration curve, according to Bradford [43].

### 3.6. Enzyme Assay

PPO activity can be monitored by oxygen consumption or spectrophotometrically using a variety of substrates such as pyrogallol, pryocatechol, 4-methylcatechol, 3,4-dihydroxyphenylacetic acid, 4-*tert*-butylcatechol, and chlorogenic acid [8]. In the current study, PPO activity was determined spectrophotometrically at 265 nm by monitoring the reaction at 25 °C for 5 min. The final concentrations in the reaction mixture were 50 mM potassium phosphate, 0.17 mM l-3,4-dihydroxyphenylalanine, 0.072 mM ascorbic acid, 0.0022 mM ethylenediaminetetraacetic acid, and 50–100 units of PPO. One unit of enzyme activity was defined as the amount of enzyme causing a 0.001 change in the absorbency per minute at pH 6.5 at 25 °C in 3 mL reaction mix.

Finally, the activity of PPO enzyme was calculated by using Equation (2), following Behzadi et al. [44],

(2) $$U=\frac{Units}{mg}enzyme=\frac{\left(\Delta A_{265\;\mathrm{nm}}\;TEST\;-\;\Delta A_{265\;\mathrm{nm}}BLANK\right)\;\times df}{0.001\times 0.1\;mL}$$

where 0.001 represents the change in *A*_265 (nm/min)_ per unit of polyphenol oxidase in a 3.0 mL reaction mixture at pH 6.5 at 25 °C and 0.1 represents the volume (in milliliters) of enzyme used in the assay.

The residual activity of PPO (%) was defined as the ratio between the enzyme activity of the untreated flour and the enzyme activity of scCO_2_-treated flour. The percentage of residual activity in the graham flour was calculated, using Equation (3) from Hojnik Podrepšek et al. [15]

(3) $$Residual\;activity\;of\;PPO\;\left(\%\right)=\frac{PPO\;activity\;of\;scCO_{2}\;treated\;flour}{PPO\;activity\;of\;untreated\;flour}\times 100$$

### 3.7. Determination of Total Phenol Content

The total phenols were determined using the protocol proposed by Škerget et al. (2005) using the Folin-Ciocalteu method [45]. Folin-Ciocalteu reagent (2.5 mL), which was prediluted 1:10 with water and 2 mL of Na_2_CO_3_ (75 g/L), was added to 0.5 mL of extract (supernatant after protein extraction), and the resulting mixture was thoroughly agitated. The sample was incubated for 5 min at 50 °C. 0.5 mL of distilled water was taken for the control sample. After cooling the samples, the absorbance was measured at 760 nm using a UV/VIS spectrophotometer (Varian Cary Probe 50, Agilent Technologies, Santa Clara, CA, USA). Phenol content was determined using gallic acid (GA) as standard and expressed as mg GA/g flour material. The analyses were performed in triplicate, where an average deviation was calculated.

### 3.8. Determination of Graham Flour Properties

To determine the influence of scCO_2_ on graham flour quality, characterization of flour particles was performed by particle size determination and SEM analysis, with the moisture content of untreated and scCO_2_-treated graham flour also determined.

#### 3.8.1. Particle Size Distribution

The particle size distribution of untreated and scCO_2_-treated graham flour was measured using a laser diffraction particle size analyzer (Analysette 22, Fritsch), using a dry analysis method within the selected size range from 0.3 to 300 µm. The sample was placed into the sample hopper and then aspirated into the apparatus. The distribution was reported as the arithmetic mean (µm), where three replicates were performed within a standard deviation of ±5 µm.

#### 3.8.2. Scanning Electron Microscopy of Graham Flour

The size and morphology of the graham flour particles were analyzed by SEM (FE, SEM SIRION, 400 NC, FEI, Hillsboro, OR, USA). The flour particle morphology and microstructural changes were observed on untreated and scCO_2_-treated graham flour, with the resulting SEM images used to study the changes in size and shape of the particles after scCO_2_-treatment.

#### 3.8.3. Moisture Content

Moisture content was measured using an HB43-S (METTLER TOLEDO, Greifensee, Switzerland) halogen moisture analyzer. This performs measurements based on the thermogravimetric principle, i.e., the moisture is determined from the weight loss of a sample dried by heating to 130 °C, within a standard deviation of ±0.2% and with duplicated measurements.

## 4. Conclusions

The aim of this study was to investigate the effects of scCO_2_ on the protein concentration and inactivation of enzyme PPO in graham flour after scCO_2_ exposure. The effects of pressure as a process parameter on protein concentration and PPO activity in graham flour were studied. The results clearly showed significant changes in protein concentration induced by scCO_2_, which fell by 20% after scCO_2_ treatment at 300 bar for 24 h, and could be ascribed to the leakage of proteins into the media. These results also indicate that significant changes in enzyme activity caused by scCO_2_ may be associated with the conformation of enzyme molecules, as evidenced by a 35% decrease in enzyme activity of PPO in graham flour after scCO_2_ exposure at 300 bar for 24 h. At the same time, the results showed that the quality of the flour remained almost the same or even improved. The current study is important as the technology of scCO_2_ was recognized as appropriate because it does not adversely affect the quality of the flour, which was proved with SEM analysis, where starch grains of graham flour after scCO_2_ treatment were not damaged and therefore, scCO_2_-treated graham flour is still suitable for use in the bakery industry for wheat end-use products. In addition, the correlation of PPO activity and total phenolic content of untreated and with scCO_2_-treated graham flour was examined. This study provided an effective method of scCO_2_ technology, which was found to be a suitable technology to inactivate the PPO enzyme, which might be a crucial factor to induce the chemical reactions resulting in undesirable darkening of flour end-use products and can thereby extend the shelf life of the graham flour.

## Figures and Tables

**Table 1:** Comparison of the particle size distribution of the graham flour and the moisture content as a function of scCO_2_ treatment.

Sample	Mean Particle Size (µm)	Moisture Content (%)
Untreated graham flour	82 ± 5	12.4 ± 1.6
scCO_2_-treated graham flour	74 ± 5	11.9 ± 1.7

**Table 2:** List of conditions that were applied in the protein extraction process.

Sample Abbreviation	Conditions	Centrifugation (rpm)
GM8000	Graham flour stored at room temperature	8000
GHL8000	Graham flour stored in a refrigerator (2–8 °C)	8000
GST8000	Graham flour stored at room temperature with the addition of glass beads	8000

## References

[1] Lin S., Gao J., Jin X., Wang Y., Dong Z., Ying J., Zhou W. (2020). Whole-wheat flour particle size influences dough properties, bread structure and in vitro starch digestibility. Food Funct..

[2] Zhai S., He Z., Wen W., Liu J., Jin H., Yan J., Zhang Y., Zhang P., Wan Y., Xia X. (2020). Genetic architecture of polyphenol oxidase activity in wheat flour by genome-wide association study. Crop Sci..

[3] Tinello F., Lante A. (2018). Recent advances in controlling polyphenol oxidase activity of fruit and vegetable products. Innov. Food Sci. Emerg. Technol..

[4] Kuddus M. (2018). Enzymes in Food Technology: Improvements and Innovations.

[5] Nirmal N.P., Benjakul S., Ahmad M., Arfat Y.A., Panichayupakaranant P. (2015). Undesirable Enzymatic Browning in Crustaceans: Causative Effects and Its Inhibition by Phenolic Compounds. Crit. Rev. Food Sci. Nutr..

[6] Debelo H., Li M., Ferruzzi M.G. (2020). Processing Influences on Food Polyphenol Profiles and Biological Activity. Curr. Opin. Food Sci..

[7] Tomás-Barberán F.A., Espín J.C. (2001). Phenolic compounds and related enzymes as determinants of quality in fruits and vegetables. J. Sci. Food Agric..

[8] Nokthai P., Lee V.S., Shank L. (2010). Molecular Modeling of Peroxidase and Polyphenol Oxidase: Substrate Specificity and Active Site Comparison. Int. J. Mol. Sci..

[9] Khan M., Ahmad K., Hassan S., Imran M., Ahmad N., Xu C. (2017). Effect of novel technologies on polyphenols during food processing. Innov. Food Sci. Emerg. Technol..

[10] Hou G.G. (2010). Asian Noodles: Science, Technology, and Processing.

[11] Zhao Y., Huang Z.-H., Zhou H.-M., Zhu K.-X., Guo X.-N., Peng W. (2020). Polyphenol oxidase browning in the formation of dark spots on fresh wet noodle sheets: How dark spots formed. Food Chem..

[12] Guo X.-N., Wu S.-H., Zhu K.-X. (2020). Effect of superheated steam treatment on quality characteristics of whole wheat flour and storage stability of semi-dried whole wheat noodle. Food Chem..

[13] Doble M., Kruthiventi A.K., Doble M., Kruthiventi A.K. (2007). CHAPTER 9-Industrial Examples. Green Chemistry and Engineering.

[14] Manzocco L., Plazzotta S., Spilimbergo S., Nicoli M.C. (2017). Impact of high-pressure carbon dioxide on polyphenoloxidase activity and stability of fresh apple juice. LWT-Food Sci. Technol..

[15] Hojnik Podrepšek G., Knez Ž., Leitgeb M. (2019). Activation of cellulase cross-linked enzyme aggregates (CLEAs) in scCO_2_. J. Supercrit. Fluids.

[16] Ferrentino G., Spilimbergo S. (2011). High pressure carbon dioxide pasteurization of solid foods: Current knowledge and future outlooks. Trends Food Sci. Technol..

[17] Zhong Q., Jin M. (2008). Enhanced Functionalities of Whey Proteins Treated with Supercritical Carbon Dioxide. J. Dairy Sci..

[18] Matsuda T. (2013). Recent progress in biocatalysis using supercritical carbon dioxide. J. Biosci. Bioeng..

[19] Benito-Román Ó., Sanz M.T., Illera A.E., Melgosa R., Benito J.M., Beltrán S. (2019). Pectin methylesterase inactivation by High Pressure Carbon Dioxide (HPCD). J. Supercrit. Fluids.

[20] Goyeneche R., Di Scala K., Roura S. (2013). Biochemical characterization and thermal inactivation of polyphenol oxidase from radish (*Raphanus sativus* var. *sativus*). LWT.

[21] Garcia-Palazon A., Suthanthangjai W., Kajda P. (2004). The effects of high hydrostatic pressure on β-glucosidase, peroxidase and polyphenoloxidase in red raspberry (*Rubus idaeus*) and strawberry (*Fragaria* × *ananassa*). Food Chem..

[22] Manzocco L., Ignat A., Valoppi F., Burrafato K.R., Lippe G., Spilimbergo S., Nicoli M.C. (2016). Inactivation of mushroom polyphenoloxidase in model systems exposed to high-pressure carbon dioxide. J. Supercrit. Fluids.

[23] Liu X., Gao Y., Xu H., Hao Q., Liu G., Wang Q. (2010). Inactivation of peroxidase and polyphenol oxidase in red beet (*Beta vulgaris* L.) extract with continuous high pressure carbon dioxide. Food Chem..

[24] Chakraborty S., Rao P.S., Mishra H.N. (2015). Kinetic modeling of polyphenoloxidase and peroxidase inactivation in pineapple (*Ananas comosus* L.) puree during high-pressure and thermal treatments. Innov. Food Sci. Emerg. Technol..

[25] Iqbal A., Murtaza A., Muhammad Z., Elkhedir A.E., Tao M., Xu X. (2018). Inactivation, Aggregation and Conformational Changes of Polyphenol Oxidase from Quince (*Cydonia oblonga* Miller) Juice Subjected to Thermal and High-Pressure Carbon Dioxide Treatment. Molecules.

[26] İçi˙er F., Yildiz H., Baysal T. (2008). Polyphenoloxidase deactivation kinetics during ohmic heating of grape juice. J. Food Eng..

[27] Cao X., Cai C., Wang Y., Zheng X. (2017). The inactivation kinetics of polyphenol oxidase and peroxidase in bayberry juice during thermal and ultrasound treatments. Innov. Food Sci. Emerg. Technol..

[28] Hu W., Zhang Y., Wang Y., Zhou L., Leng X., Liao X., Hu X. (2011). Aggregation and Homogenization, Surface Charge and Structural Change, and Inactivation of Mushroom Tyrosinase in an Aqueous System by Subcritical/Supercritical Carbon Dioxide. Langmuir ACS J. Surf. Colloids.

[29] Yadav D., Patki P., Sharma G.K., Bawa A. (2007). Effect of microwave heating of wheat grains on the browning of dough and quality of chapattis. Int. J. Food Sci. Technol..

[30] Kushnirov V.V. (2000). Rapid and reliable protein extraction from yeast. Yeast.

[31] Lu Y., Chae M., Vasanthan T., Bressler D.C. (2020). The potential of fiber-depleted starch concentrate produced through air currents assisted particle separation of barley flour in bio-ethanol production. Bioresour. Technol..

[32] Liao H., Zhong K., Hu X., Liao X. (2019). Effect of high pressure carbon dioxide on alkaline phosphatase activity and quality characteristics of raw bovine milk. Innov. Food Sci. Emerg. Technol..

[33] Kamat S.V., Beckman E.J., Russell A.J. (1995). Enzyme Activity in Supercritical Fluids. Crit. Rev. Biotechnol..

[34] Marszałek K., Doesburg P., Starzonek S., Szczepańska J., Woźniak Ł., Lorenzo J.M., Skąpska S., Rzoska S., Barba F.J. (2019). Comparative effect of supercritical carbon dioxide and high pressure processing on structural changes and activity loss of oxidoreductive enzymes. J. CO2 Util..

[35] Marszałek K., Krzyżanowska J., Woźniak Ł., Skąpska S. (2017). Kinetic modelling of polyphenol oxidase, peroxidase, pectin esterase, polygalacturonase, degradation of the main pigments and polyphenols in beetroot juice during high pressure carbon dioxide treatment. LWT-Food Sci. Technol..

[36] Solaesa Á.G., Villanueva M., Beltrán S., Ronda F. (2019). Characterization of Quinoa Defatted by Supercritical Carbon Dioxide. Starch Enzymatic Susceptibility and Structural, Pasting and Thermal Properties. Food Bioprocess Technol..

[37] Kang S.-W., Rahman M.S., Kim A.-N., Lee K.-Y., Park C.-Y., Kerr W.L., Choi S.-G. (2017). Comparative study of the quality characteristics of defatted soy flour treated by supercritical carbon dioxide and organic solvent. J. Food Sci. Technol..

[38] Hironaka K., Ishibashi K.-I., Koaze H., Shirasaka H., Matsuda K., Sato T., Kojima M., Mori M., Tsuda S., Takada A. (2006). Effects of polyphenol content, polyphenol oxidase activity and pH on blackspot bruise of Japanese potato varieties. Food Preserv. Sci..

[39] Esmaeili N., Ebrahimzadeh H., Abdi K. (2017). Correlation between polyphenol oxidase (PPO) activity and total phenolic contents in *Crocus sativus* L. corms during dormancy and sprouting stages. Pharmacogn. Mag..

[40] Brown Z.K. (2010). The Drying of Foods Using Supercritical Carbon Dioxide.

[41] Lapčíková B., Burešová I., Lapčík L., Dabash V., Valenta T. (2019). Impact of particle size on wheat dough and bread characteristics. Food Chem..

[42] Primožič M., Čolnik M., Knez Ž., Leitgeb M. (2019). Advantages and disadvantages of using SC CO_2_ for enzyme release from halophilic fungi. J. Supercrit. Fluids.

[43] Bradford M.M. (1976). A rapid and sensitive method for the quantitation of microgram quantities of protein utilizing the principle of protein-dye binding. Anal. Biochem..

[44] Behzadi R., Sadeghizadeh M., Movahedi A.A.M., Sabouri A.A., Sattari T.N. (2013). Gain of human tyrosinase DOPA oxidase activity in artificial M374 Asp mutant. Life Sci. J..

[45] Škerget M., Kotnik P., Hadolin M., Hraš A.R., Simonič M., Knez Ž. (2005). Phenols, proanthocyanidins, flavones and flavonols in some plant materials and their antioxidant activities. Food Chem..

